# Integrative metabolomics using untargeted UHPLC-MS/MS and chemometrics identifies optimal maturity stage of *Moringa oleifera* leaves from Kuala Terengganu, Malaysia

**DOI:** 10.7717/peerj.20938

**Published:** 2026-03-18

**Authors:** Ummi Kalthum Azlan, Hadi Azamuddin Abd Hakim, Pei Lou Wong, Noor Hanini Che Lah, Chenyu Zhou, Nur Irlia Sofea Mohammad Zamani, Xiaohui Tong, Rongchun Han, Norazlan Mohmad Misnan, Murni Nazira Sarian, Emelda Rosseleena Rohani, Ahmed Mediani, Faidruz Azura Jam, Hamizah Shahirah Hamezah

**Affiliations:** 1 Institute of Systems Biology (INBIOSIS), Universiti Kebangsaan Malaysia, UKM Bangi, Selangor, Malaysia; 2 Sunway Biofunctional Molecules Discovery Centre, Faculty of Medical and Life Sciences, Sunway University, Petaling Jaya, Selangor, Malaysia; 3 Faculty of Medicine, Manipal University College Malaysia, Jalan Batu Hampar, Bukit Baru, Malacca, Malaysia; 4 School of Life Sciences, Anhui University of Chinese Medicine, Hefei, China; 5 School of Pharmacy, Anhui University of Chinese Medicine, Hefei, China; 6 Herbal Medicine Research Centre, Institute for Medical Research, National Institutes of Health, Shah Alam, Selangor, Malaysia

**Keywords:** *Moringa oleifera*, Maturity stages, Antioxidant, Metabolomics, Polyphenols, Flavonoids

## Abstract

**Background:**

*Moringa oleifera* is a medicinal plant rich in bioactive compounds. Native to Southeast Asia, it thrives in tropical climates like Malaysia. Maturity stages of *M. oleifera* leaves might substantially affect the effectiveness of therapy; hence, optimizing the bioactive phytoconstituents is crucial. Liquid chromatography-mass spectrometry(LC-MS)-based metabolomics is essential to (i) evaluate differences in phytochemical composition and bioactivities across leaf maturity, (ii) profile and model leaf metabolome across maturity stages using untargeted ultra-high performance liquid chromatography tandem mass spectrometry (UHPLC-MS/MS) for key metabolites annotation, and with multivariate data analysis (MVDA) to reveal maturity-linked clusters and discriminant metabolites, and (iii) integrate multivariate regression to relate metabolite signatures to bioassays and nominate optimal maturity stages.

**Methods:**

*M. oleifera* leaves were harvested at different maturity stages (day-30, 45, and -60) from Kuala Terengganu, Malaysia. Aqueous ethanolic extracts were subjected to bioactivity assays, including total phenolic (TPC) and flavonoid contents (TFC), 2,2-diphenyl-1-picrylhydrazyl (DPPH) radical scavenging, ferric reducing power test (FRAP) antioxidant activity, and acetylcholinesterase (AChE) inhibition. Metabolomics profiling was conducted, and the correlation of phytochemicals to bioactivities was performed by multiple MVDA models.

**Results:**

Day-60 leaf extracts exhibited higher TPC and TFC compared to younger stages, with strong antioxidant activity as indicated by elevated DPPH and FRAP values. In contrast, AChE inhibition was highest in day-30 extracts. Metabolomics profiling with chemometrics integration using MVDA identified and tentatively annotated 27 metabolites with predominantly flavonoids (59%), phenolic acids (19%), glucosinolates (7%), and minor components including coumarins, folates, and alkaloid-like compounds. Principal component analysis (PCA) and partial least squares (PLS) biplot revealed distinct metabolic clustering, with day-60 extract formed clear separated cluster, determined by accumulation of quercetin rutinoside, chlorogenic acid, kaempferol derivatives, and procyanidin B2. PLS biplot demonstrated that quercetin derivatives, chlorogenic acid, and procyanidin B2 were positively associated with antioxidant indicator, while coumarin and folic acid aligned with AChE inhibition. Unknown metabolites indicated by chromatogram peak area with high Variable Importance in Projection (VIP) scores (>1) also contributed to bioactivity trends and separation, lead to potential of unannotated phytochemicals.

**Conclusion:**

This study found that maturity stage influences the phytochemistry and bioactivities of *M. oleifera* leaf. Chemometrics integration of metabolomics analysis with bioassays shows that maturity stages drive distinct metabolite clustering and bioactivities, with day-60 yielding the highest phenolic, flavonoid, and antioxidant capacity (DPPH/FRAP), while day-30 exhibits the strongest AChE inhibition, thereby, defining maturity-specific optimal harvests.

## Introduction

Plant-based products are a significant initiative in medical research, exploiting the potential properties possessed by bioactive compounds naturally occurring in plants. Extensive research has focused on integrating plant products into the daily diet and harnessing their potential efficacy in developing safe medicines through synthetic strategies, offering new perspectives for disease prevention and treatment (Adarthaiya & Sehgal, 2024). Malaysia is famous for its rich biodiversity, and for many generations, various herbal plants in Malaysia have been used in traditional medicine. One highly regarded Malaysian plant known for its health benefits is *Moringa oleifera*. Native to India and widely cultivated in South Asia, *M. oleifera* is distinguished by its slender leaves, white flowers, and elongated flat pod fruits (Saucedo-Pompa et al., 2018).

*M. oleifera* is commonly referred to as the “miracle tree,” where this designation stems from the plant’s remarkable nutritional profile. Every part of the plant especially the leaves and seeds have been recognized to contain a variety of bioactive compounds, including vitamins, carotenoids, polyphenols and flavonoids (Azlan et al., 2022). These compounds greatly enhance the potential of *M. oleifera* as a nutritional supplement, nutraceutical, and pharmaceutical product. Furthermore, the seeds, flowers, and bark of *M. oleifera* are rich in various phytochemical compounds. Notably, the seeds contain glucosides and isothiocyanates, which exhibit significant anti-cancer, anti-inflammatory, and antibacterial properties (Shahbaz et al., 2024). Previous studies have shown that the concentration of bioactive components and protein expression in leaves and seeds differ in diverse stages of maturity, especially polyphenols and flavonoids. The leaves of mature plants are rich in higher concentrations of these active components and thus have greater medicinal potential (Garcia et al., 2019; Proestos & Varzakas, 2017).

Antioxidant activity is a vital component of the pharmacological value of *M. oleifera* leaves, which is mainly attributed to its abundance of polyphenols and flavonoids. Antioxidants can protect cells from damage by scavenging free radicals and reducing oxidative stress. Studies have shown that mature leaves exhibit higher activity in scavenging 2,2-diphenyl-1-picrylhydrazyl (DPPH) radicals, 2,2′-azino-bis (3-ethylbenzothiazoline-6-sulfonic acid) (ABTS) cationic radicals, and superoxide anions (Sreelatha & Padma, 2009). This antioxidant activity makes *M. oleifera* a potential candidate for application in the prevention of oxidative damage related diseases such as cardiovascular disease, cancer, and diabetes. Previous studies also have revealed the potential neuroprotective properties of *M. oleifera* against neurodegenerative diseases (Ghimire et al., 2021; Pandey et al., 2020). Its cholinesterase inhibition, anti-amyloid aggregation and antioxidant activity have been used to alleviate the symptoms of Alzheimer’s disease (Adebayo et al., 2021; Azlan et al., 2023; Nwidu et al., 2018).

Metabolomics studies of *M. oleifera* through liquid chromatography-mass spectrometry (LC-MS) techniques have revealed the diversity of secondary metabolites and their associated biological activities (Saucedo-Pompa et al., 2018). The metabolites produced by *M. oleifera* leaves during growth included a range of amines, amino acids, phenols, and lipids. These secondary metabolites are not only closely related to plant growth and development but also play an important role in the antioxidant and anti-inflammatory properties of plants (Vergara-Jimenez, Almatrafi & Fernandez, 2017). Metabolomics studies provide a more comprehensive understanding of the nutritional value and potential pharmacological properties of *M. oleifera* leaves at various maturation stages, thus providing scientific basis for the development of *M. oleifera*-based products for different purposes.

However, the contents of bioactive compounds and secondary metabolites in leaves of *M. oleifera* at different maturity stages, as well as the changes of main phytochemical components, are rarely published in recent years. As plant-based products are one of the promising approaches to utilize natural bioactive compounds for medical research, the lack of age-dependent phytochemical and bioactivity profiling of *M. oleifera* leaves has limited its potential in functional food and pharmaceutical applications. Therefore, this study systematically investigates the phytochemical and bioactivity profiles of *M. oleifera* leaves at different maturity stages. By comparing the metabolite composition and their associated biological activities across maturity levels, this work aims to provide a more robust scientific basis for the application of *M. oleifera* in the development of health supplements and pharmaceutical products.

## Materials and Methods

### Chemicals and reagents

The chemicals and reagents used in this study included quercetin, quercetin-3-rutinoside (rutin), p-coumaric acid, caffeic acid, were purchased from Sigma Aldrich Co. (Darmstadt, Germany). Kaempferol and kaempferol-3-glucpside (astragalin) were obtained from ChemFaces (Wuhan, China). All solvents used for the preparation of the sample and MS analysis were (UHPLC-MS/MS) graded solvent purchased from Fisher Chemical (Geel, Belgium).

### Collection and preparation of *M. oleifera* leaves across maturity stages

Leaf samples were obtained from the *M. oleifera* field owned by MR Moringa Sdn. Bhd. located in Kuala Terengganu, Terengganu, Malaysia. The species was verified by botanist Dr. Shamsul bin Khamis, and a voucher specimen (ID022/2022) was deposited at the Universiti Kebangsaan Malaysia’s Herbarium (UKMB). The leaves were plucked at pre-defined maturity stages, which are on day-30 (young/expanding), day-45 (mid-mature), and day-60 (fully mature). For standardization, all selected trees (same planting source and comparable age class) were pruned, and day counting started immediately post-pruning. Leaflets were pooled from two trees (trees ≥ 10 m apart- to reduce individual-tree effects) for one replicate, and each maturity stage was allocated for three biological replicates (two trees per replicate × three biological replicates × three maturity stages). Sampling was performed in the morning window using clean shears with a consistent cutting strategy; only fully expanded, undamaged leaflets from mid-canopy were taken, and diseased or damaged tissues were excluded. Collected leaflets were cleaned using water and stored overnight at −80 °C. The freeze-drying process was applied to the frozen leaves and kept for 3 days to obtain a constant mass.

### Preparation of extracts using ethanol solution

Before the extraction process, each *M. oleifera* leaves were freeze-dried and grounded into a fine powder using a laboratory blender. Next, 4 g of each leaf sample (18 biological replicates) were extracted using 100 ml of 80% ethanol for 1 h in an ultrasonicator machine (90 W, 53 kHz). The mixture was placed in a vacuum funnel for 30 min to separate the supernatant and precipitate. The supernatants were collected and concentrated using a rotary evaporator. Six separate extractions were performed to obtain six biological replicates of the samples for each stage of leaf maturity (Makkar & Becker, 1997).

### Total flavonoid content

The determination of total flavonoid content (TFC) was determined based on the aluminium chloride colorimetric assay method (Elin Novia, Berna & Rani, 2018). Sample extracts of *M. oleifera* leaves were dissolved with ethanol (1 mg/ml). A total of 50 µl of the extracts (1 mg/ml) was added along with 10 µl of 10% aluminium chloride (AlCl_3_) and followed by 150 µl of 96% ethanol. A total of 10 µl of 1M sodium acetate was added into the 96-well microplate. The mixture was incubated for 40 min at room temperature and protected from light. The absorbance was measured using a UV-Vis microplate reader at a wavelength of 415 nm, as the reaction between Al^3+^ ions and bioactive compounds in the sample produces a yellow color detectable by the reader. A standard curve using quercetin was generated using concentrations of 20, 40, 60, 80, and 100 µg/ml in ethanol. The extract yield was quantified and expressed as mg of quercetin equivalent (QE) per gram of leaf extract.

### Total phenolic content

The determination of the total phenolic content (TPC) was carried out using Folin-Ciocalteu assay (Bristy et al., 2022). Samples were prepared by dissolving the extract in ethanol (1 mg/ml). A total of 30 µl of sample and 120 µl of Folin-Ciocalteu reagent were mixed into microtiter plates of 96-wells and incubated for 5 min. Then, 150 µl of 7.5% sodium carbonate was added to the mixture. The plate was incubated in dark for 2 h and tested for absorption at 765 nm using a microplate reader. The reaction of Folin-Ciocalteu reagent with phenolic compounds in the sample results in a colour change from colourless to dark blue, with the intensity of the blue color being directly proportional to the concentration of phenolic compounds present. Each sample was analyzed in triplicate. A standard curve of gallic acid was generated to calculate the total phenolic content and the results of the extract were expressed as mg of gallic acid equivalent (GAE) per gram of extract.

### Free radical scavenging activity test

Free radical scavenging activity of *M. oleifera* leaves extract was calculated based on hydrogen donation or free radical capacity using the free radical reducing test (DPPH) (Blois, 1958). The fraction of free radical scavenging activity test (DPPH) was evaluated based on the last method described (Li et al., 2022). The preparation of the standard stock solution of 0.1 mM DPPH solution was freshly prepared for each use. A 100 mL of volumetric flask was filled with 4 mg of DPPH powder, which was then diluted with ethanol. For the assay, 20 µl of the sample was mixed with 180 µl DPPH solution in a 96-well microplate. The mixture was incubated for 30 min in the dark at room temperature, with three replicates per sample. After incubation, the absorbance was measured using a UV-Vis microplate reader at a wavelength of 517 nm. For the blank sample, 20 µl of ethanol were mixed with 180 µl of DPPH solution and measured at the same wavelength. Ascorbic acid was used as a positive control and the analysis of this test was performed in triplicate. Antioxidant activity was expressed as IC_50_, representing the sample concentration required to scavenge 50% of free radicals. The calculation of IC50 for the sample and the percentage of free radical scavenging activity were calculated according to the following formula.

Free radical scavenging activity % = [(AB − AS)/AS] × 100, where AB is the absorbance of the blank and AS denotes the absorbance of the test sample.

The percentage of free radical scavenging activity from three extracts of different maturity levels was used to produce a plot for calculating the IC50 value of the *M. oleifera* samples including the positive control (Li et al., 2022).

### Ferric reducing power test

The antioxidant activity for each sample was determined using the Benzi and Strain method with slight modifications (Benzie & Strain, 1996). The ferric reducing power test (FRAP) working reagent was prepared by combining three components: 300 mM acetate buffer, pH 3.6 (CH_3_OOH/CH_3_COONa), 10 mM solution of 2,4,6-tripyridyl-1,3,5-triazine (TPTZ) in 40 mM hydrochloric acid (HCl) and 20 mM solution of FeCI_3_•6H_2_O in a ratio of 10:1:1. A solution of 300 mM acetate buffer, pH 3.6 was prepared by dissolving 0.31 g sodium acetate trihydrate (CH_3_COONa•3H_2_O) in 1.6 mL glacial acetic acid. The mixture was diluted with 100 mL of distilled water. A 40 mM HCl solution was prepared prior to the preparation of the TPTZ solution by diluting 1.67 mL of the 12 M HCl solution with 500 mL of distilled water. For the TPTZ solution, 0.1562 g of TPTZ powder was diluted in 40 mM HCl solution and for the preparation of 20 mM FeCI3•6H_2_O (ferric (III) chloride hexahydrate) solution was prepared by mixing 0.2703 g of FeCI3•6H_2_O powder with distilled water until the mixture was completely dissolved and filled to 50 mL. The preparation of these three ingredients produces a fresh FRAP solution that was heated to 37 °C prior to use. For antioxidant activity analysis, 50 µl of the extract sample was mixed with 1,950 µl of fresh FRAP solution. After incubating the mixture for 30 min, 200 µl was transferred to a 96-well microplate for absorbance measurements using a UV-Vis microplate reader at a wavelength of 595 nm. Trolox was used as the standard.

### Anticholinesterase determination test

The method of inhibition of the enzyme acetylcholinesterase (AChE) was carried out with slight modifications using spectrophotometric techniques (Ahmad et al., 2018). This method used the Electric Eel AChE enzyme (type-VI-S Electric Eel acetylcholinesterase, EC 3.1.1.7, Sigma–Aldrich, St. Louis, MO, USA) and acetylthiocholine iodide substrate (Sigma-Aldrich, Steinheim, Germany). In brief, 125 μl of DTNB/Ellman Reagent (5,5-dithio-bis-(2-nitrobenzoic acid)) (Sigma–Aldrich, Steinheim, Germany) (50 mM Tris-HCl, pH 8, 0.1 M NaCl, 0.02 M MgCl_2_.6H_2_O), 25 μl of AChE (0.2 U/ml), 25 μl of test solution in DMSO (dimethyl sulfoxide), and 50 μl of buffer (50 mm tris-HCl, pH 8, 0.1% BSA) was mixed and incubated at 25 °C for 30 min. For control experiments, ethanol (25 μl) was added to replace the compound test solution. A total of 25 μl of acetylthiocholine iodide (0.25 mmol/l) was added to initiate the reaction, resulting in a final volume of 250 μl. The formation of the 5-thio-2-nitrobenzoate anion from hydrolysis of the enzyme acetylthiocholine iodide was controlled based on 412 nm absorption using a 96-well microplate reader (Model 680; Biorad Inc., Hercules, CA, USA).

Data collected at specific time points divided into 20-s increments over the first 180 s, were used to calculate the reaction rate. The percentage of inhibition by AChE was determined based on the ratio of the reaction rate of the test sample to the control blank (DMSO in tris-HCl buffer, pH 8.0) using the formula ((E − S)/E × 100), where E is the enzyme activity with DMSO, and S is the enzyme activity with the test sample. The experiment was conducted three times. Donepezil, a drug that slows the degeneration rate of Alzheimer’s disease, was used as a standard.

### Metabolite profiling and quantification of plant bioactive compounds using ultra-high performance liquid chromatography-tandem mass spectrometry

Ultra-high performance liquid chromatography-tandem mass spectrometry (UHPLC-MS/MS) was used to identify bioactive compounds in *M. oleifera* leaves extracts of different maturity stage group. The chromatographic separation was performed with the Thermo Scientific Dionex Ultimate 3000 Series RS system (Thermo Fisher Scientific, Waltham, MA, USA), operated *via* Chromeleon 7.2 software (Dionex Softron GMbH, Germering, Germany). Analyses were carried out using an ACQUITY UPLC® BEH C18 pre-column (2.1 × 100 mm, 1.7 μm; Waters, Milford, MA, USA) fitted with a VanGuard BEH C18 pre-column (2.1 × 5 mm, 1.7 m) kept at 40 °C. The mobile phase consisted of solvent A (0.1% v/v formic acid in water) and solvent B (0.1% v/v formic acid in acetonitrile solution). A gradient elution was applied as follows: 5% solvent B for 0–5 min, 5–95% solvent B from 5.0 to 17.0 min, 95% solvent B for 17.0 to 18.5 min, and re-equilibration to 5% solvent B for 18.5 to 20.0 min. The flow rate of was set at 0.3 mL min^−1^, and the injection volume was 1 µL.

The mass spectrometric detection was conducted using an LTQ-XL ion trap mass spectrometer (Thermo Fisher Scientific, San Jose, CA, USA) equipped with an electrospray ionization (ESI) source operating in negative ion mode. This ionization was chosen due to the better ionization profiles observed in the negative ion mode. Source conditions were: 3.5 kV; Capillary voltage: 14.96 V; Source temperature: 122.53 °C: sheath gas flow rate 34.98; auxiliary gas flow rate 15.03. The spectrum data type was centroid with a scan range from m/z 100 to 1,500. Data were analysed using the Xcalibur Software package (Thermo Fisher Scientific, Waltham, MA, USA) and identification of corresponding peaks were accomplished by comparing the deprotonated molecular ions and fragmentation patterns to literature search and mass spectral databases such as KNApSAcK, Metabolomics Workbench, Human Metabolome Database (HMDB), PubChem, GNPS and MassBank.

MS-DIAL software (RIKEN version 4.19.2218) was used to aid with spectral data processing and determination of peak area for representation of relative abundance or intensity of detected metabolites. Raw files were centroided (negative ESI), converted to mzML with ProteoWizard MSConvert and processed in MS-DIAL. As the data were acquired on an ion trap (unit-mass) analyser, absolute mass tolerances appropriate to that platform were used; MS^1^ 0.01 Da at the peak finding/prefilter stage to stabilize chromatographic peak integration (without implying HRMS-level mass accuracy), MS^2^ 0.05 Da for the fragment matching, and MS^1^ 0.015 Da/RT 0.05 min for alignment. The analytical window was 0–20 min, m/z 100–1,500 for MS^1^/MS^2^. Peak-picking parameters were minimum peak height 1.0 × 10^3^ a.u., mass slice width 0.1 Da, with smoothing linear weighted moving average (level 3 scans), and minimum peak width 5 scans. Alignment was then performed with gap filling enabled. Blank-based filtering and presence-filtering (feature-detected in ≥2 of 3 replicates per group) were applied to reduce artefacts, before exporting peak areas for relative quantification and MVDA.

For library matching and identification confidence, Metabolomics Standard Initiative (MSI) levels were applied. Identification was performed using the MSMS-Public_all-neg-VS19.msp library in MS-DIAL with RT tolerance 2.0 min, MS^1^ 0.01 Da, MS^2^ 0.05 Da. MS-DIAL total scores were reported where available, and spectral similarities for fragments matched from online mass spectral libraries were determined as MS/MS fragment ions. Putative annotations required a match score ≥70% and ≥2 diagnostic fragments/neutral losses consistent with the proposed adduct/chemistry. MSI-1 level was classified as metabolites confirmed with authentic standard (same-method RT ± 0.2 min and diagnostic MS/MS fragments match), while MSI-2 level is a library match structure that meets prior criteria, and MSI-3 level is reported when positional isomers could not be uniquely resolved.

Quantifications of the selected compounds were performed based on International Council for Harmonisation (ICH) Q2 (R2) guidelines (Borman & Elder, 2005), evaluating key validation parameters that include specificity, accuracy, linearity, precision (repeatability, intra- and inter-day), as well as the limit of detection (LOD), and limit of quantification (LOQ). Six bioactive polyphenolic compounds of *M. oleifera* which are kaempferol, quercetin, kaempferol 3-glucoside (astragalin), quercetin 3-rutinoside (rutin), p-coumaric acid, and caffeic acid, were also quantified by UHPLC-MS/MS. Commercialised standards for all targeted bioactive compounds were prepared in absolute ethanol, and serial dilutions were performed respectively at a starting concentration of 1 mg/ml (five dilutions; 1.0, 0.5, 0.25, 0.125, 0.0625 mg/ml). All solutions were stored at 4 °C prior to use (Ahmed et al., 2021).

### Multivariate data analysis using SIMCA

Multivariate Data Analysis (MVDA) was performed using SIMCA 18 (Sartorius Stedim Data Analytics AB, Umeå, Sweden) to investigate the metabolic differences among leaf extracts from different age groups and to explore their associations with biological activities. Peak area intensities obtained from MS-DIAL output were used as relative quantification of detected metabolites. The metabolite dataset (X-variables) was mean-centered and scaled using pareto scaling to balance the contribution of both high- and low-abundance metabolites. Principal component analysis (PCA) was carried out to observe natural clustering patterns, identify potential outliers, and assess overall variance in the dataset without any class supervision.

In addition, partial least squares (PLS) biplot was used to evaluate the correlations of bioactivity assay data, such as DPPH radical scavenging activity, FRAP reducing power, and acetylcholinesterase (AChE) inhibition as Y-variables with the metabolite profiles. Model performance was evaluated using seven-fold cross-validation, with R^2^X, R^2^Y, and Q^2^ values used to assess the explained variance and predictive accuracy. Permutation testing (*n* = 200) was conducted to assess model reliability and avoid overfitting. Discriminating metabolites were identified based on Variable Importance in Projection (VIP) scores >1.0 and visualized through PLS biplot and score plot. Metabolites data were examined for their potential biological relevance in age-related metabolic shifts and their contribution to the observed bioactivities.

## Results

### Extraction of *M. oleifera* leaves

The ethanolic extraction of *M. oleifera* leaves produced a crude yield of approximately 10.6% (w/w), equivalent to ~1.13 g extract per 100 mL of 80% ethanol solvent. This indicates that efficient recovery of soluble phytochemicals under the selected extraction conditions.

### TPC and TFC

**Figure 1:**
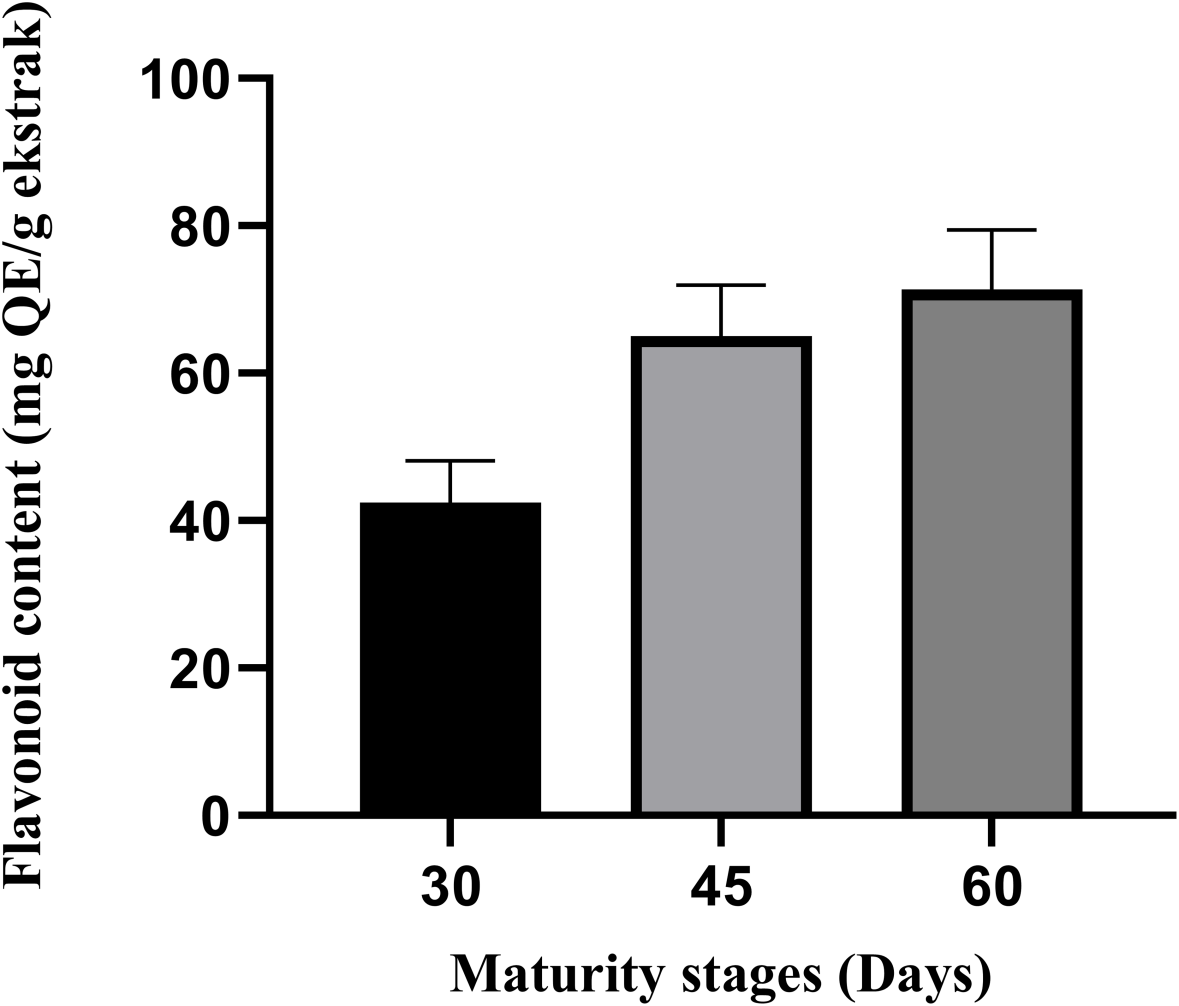
The total flavonoid content of *M. oleifera* leaves ethanolic extract. The data are represented as mean ± SEM (*n* = 3).

**Figure 2:**
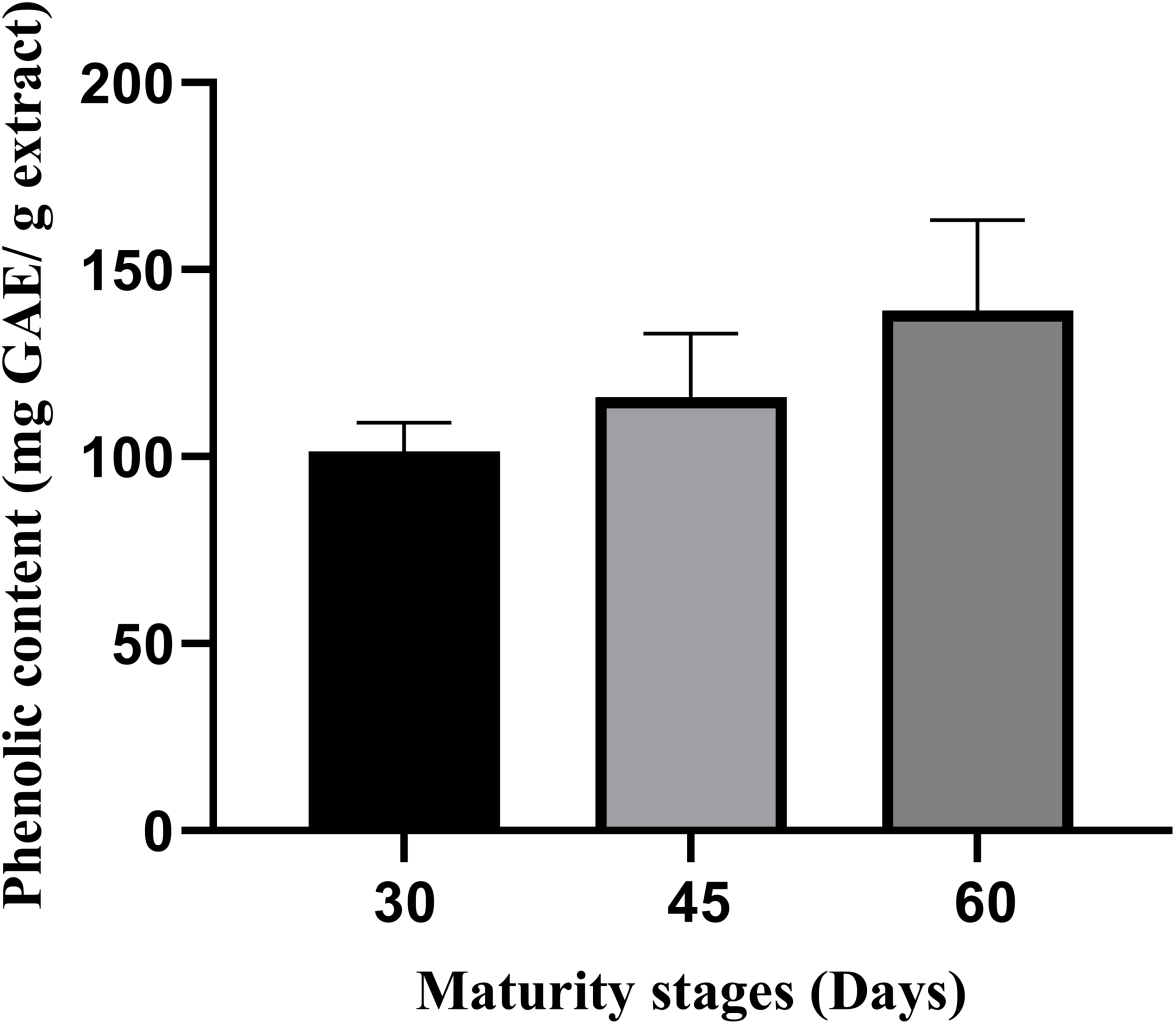
The total phenolic content of *M. oleifera* leaves ethanolic extract. The data are represented as mean ± SEM (*n* = 3).

The total flavonoid and phenolic contents (TFC, TPC) were descriptively compared to highlight the maturity trends. Although TFC and TPC values increased linearly with leaf maturity as observed in Figs. 1 and 2, these differences were not statistically significant (*p* > 0.05). This likely reflects the limited statistical power (*n* = 3) despite both assays demonstrated increasing pattern from day-30 to day-60 maturity stages. The TPC value on average is between 101.3 and 139.1 mg GAE/g extract, and the TFC value is between 42.5 and 71.3 mg QE (quercetin equivalent)/g extract (Figs. 1, 2). On average, the leaves of the 60^th^ day showed the highest total phenolic and flavonoid content (139.1 mg; 71.3 mg) compared to the 45^th^ and 30^th^ days. Furthermore, the leaves of the 30^th^ day showed the lowest total phenolic and flavonoid content (101.3 mg; 42.5 mg) compared to day 45 and day 60.

### DPPH and FRAP

Table 1 presents the antioxidant activity of DPPH for *M. oleifera* leaf extract at different stages of maturity. DPPH assay tests have been performed with both the hydrogen atom donation (HAT) mechanism as well as single electron transfer (Liang & Kitts, 2014). The IC50 value of the day-60 leaves showed the highest value, followed by day-45 which is the second highest and day-30 with the lowest IC50 value. One-way analysis of variance test (ANOVA) showed that the antioxidant activity of *M. oleifera* leaves were significantly different (*p* < 0.05) with the stages of leaf maturity. Analysis of the Tukey test was able to provide data for significantly high differences in DPPH scavenging activity on day-45 when compared to day-60 (*p* < 0.001) while significantly high differences were also found on day-30 when compared to day-45 (*p* < 0.05).

**Table 1:** IC_50_ values for DPPH test of *M. oleifera* leaf extract at different stages of maturity.

Group	IC_50_ value (mg/mL)
Standard (Ascorbic acid)	0.047 ± 0.006
Day-30	1.027 ± 0.146
Day-45	0.813 ± 0.097
Day-60	0.493 ± 0.010

**Note:**

The data presented is the mean ± SEM (*n* = 3).

**Figure 3:**
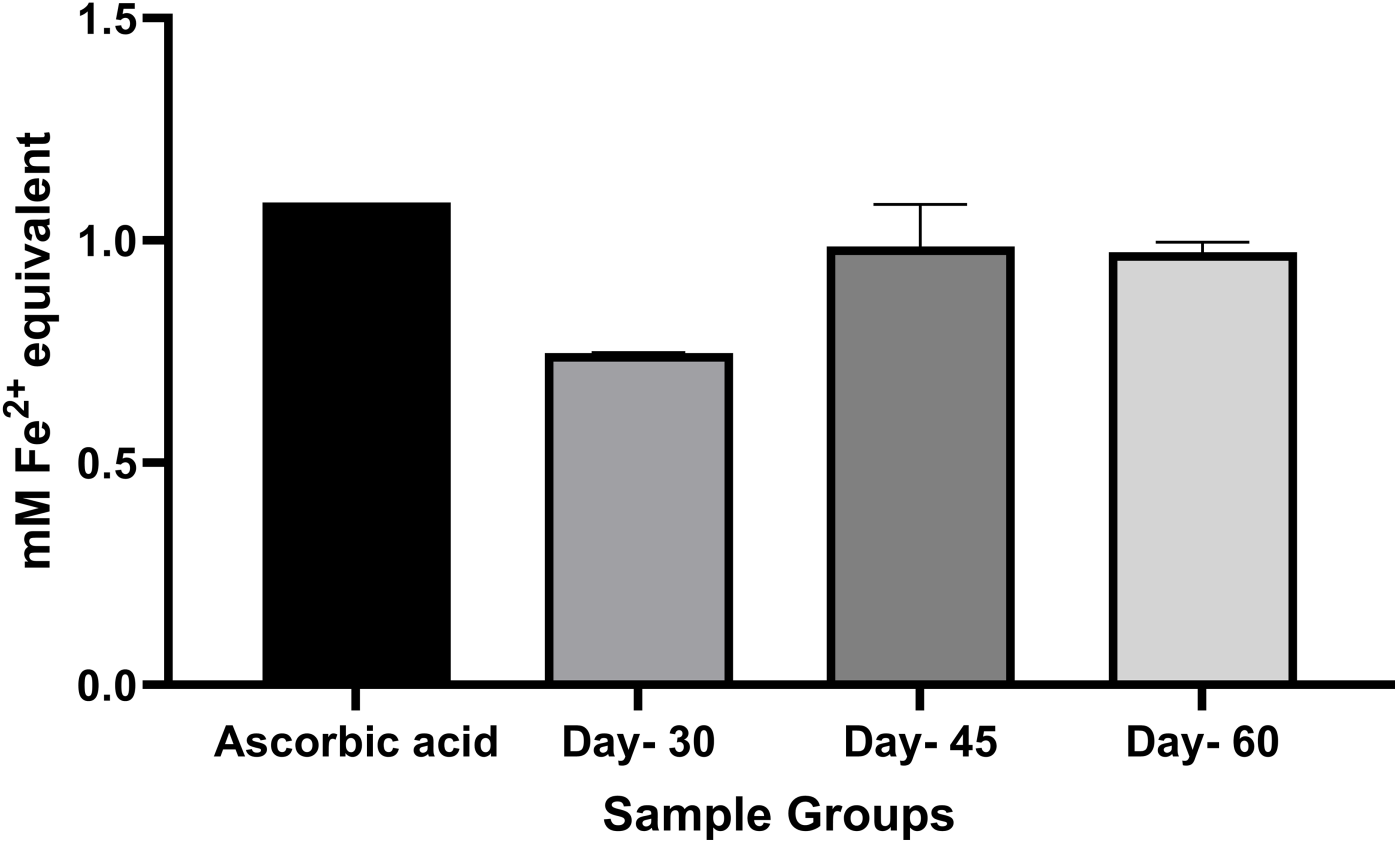
Antioxidant activity of ferric reducing power test in *M. oleifera* leaves extract between the stages of maturity. The data are represented as mean ± SEM (*n* = 3).

The ferric reducing power test of *M. oleifera* leaf extract was measured by the ability of the extract to donate electrons and reduce ferric ions (Fe^3+^) to ferrous ions (Fe^2+^), with absorbance read at 595 nm. The higher the absorbance, the higher the concentration of Fe^2+^ ions, indicating greater reducing (antioxidant) activity. Figure 3 shows the antioxidant activity of ferric reducing power test in *M. oleifera* leaf extract based on maturity stages and ascorbic acid standard. A one-way analysis of variance (ANOVA) test was performed to compare the differences between the three maturity levels of leaf extracts. The leaves in day-30 showed the lowest concentration of (Fe^2+^) (mM) while day-45 and day-60 had almost the same concentration of (Fe^2+^) (mM). There were no significant differences (*p* > 0.05) in ferric reducing power between the three maturity levels of *M. oleifera* leaf extract. The statistical differences across the stage maturity group of DPPH and FRAP assays were tabulated in Table 2.

**Table 2:** Statistical comparison of DPPH, FRAP, and AChE inhibition assays of *M. oleifera* leaf extracts at different maturity stages.

Bioactivity assays	Day-30 (mean ± SD)	Day-45 (mean ± SD)	Day-60 (mean ± SD)	ANOVA (*p*-value)
DPPH (%)	48.5587 ± 9.6788^b^	60.6565 ± 10.7033^ab^	87.2935 ± 1.6986^a^	0.0038*
FRAP (µmol FeSO_4_ equiv./g)	0.8077 ± 0.1048^a^	0.8588 ± 0.2401^a^	0.9734 ± 0.0383^a^	0.514, ns
AChE inhibition (%)	36.8087 ± 0.8377^a^	17.8189 ± 0.9580^b^	10.5715 ± 0.7491^c^	8.77 × 10^−8^*

**Notes:**

Values are mean ± SD (*n* = 3). One-way ANOVA with Tukey’s HSD *post-hoc* test was used; different subscript letters indicate significant pairwise differences (*a > b > c; p* < 0.05), identical letters denote no difference. For DPPH, day-60 > day-30 is significant, and day-45 is intermediate; FRAP showed no difference (*p* = 0.514; all a); AChE inhibition significantly decreased with maturity (day-30 > day-45 > day-60).

* Indicates significance (*p* < 0.05), ns = not significant.

### Acetylcholinesterase determination test

**Figure 4:**
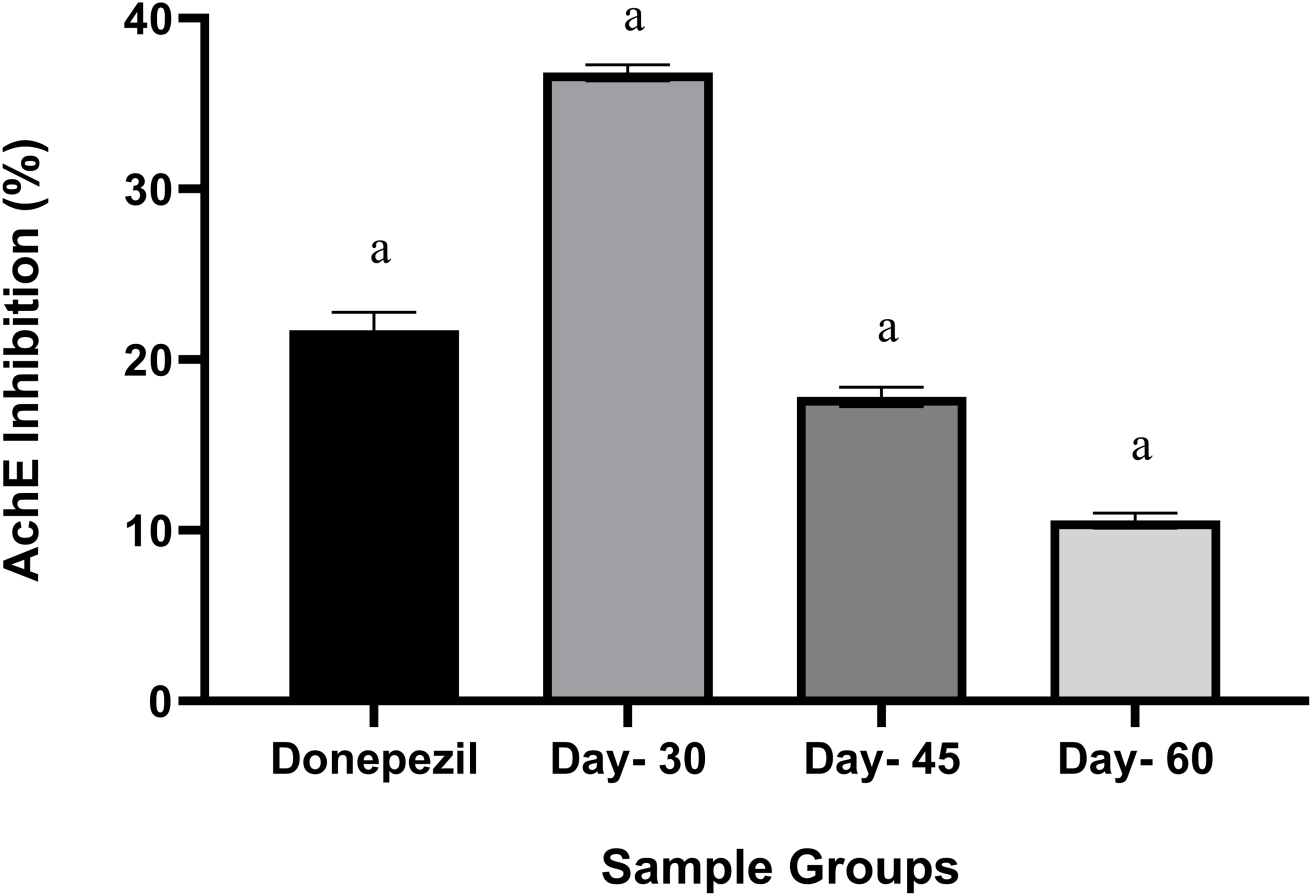
Anticholinesterase (AChE) inhibition activity by *M. oleifera* leaves ethanolic extract. The data were calculated using the percentage of AChE inhibition using donepezil as a standard. The data are represented as mean ± SEM (*n* = 3). Symbol a indicated *p* < 0.001 compared to the day-30.

*M. oleifera* leaf extracts showed significant AChE inhibitory activity with different stages of maturity (Fig. 4). Leaf extract on day-30 showed the highest inhibition of AChE (36%) and was higher than standard Donepezil (21%). A one-way analysis of variance (ANOVA) test was performed to compare the differences between the three stages of leaf extract maturity. There were significantly higher differences in the leaves of day-30 when compared to day-60 (*p* < 0.001) and the same with the leaves of day-30 when compared to day-45 (*p* < 0.001) (Table 2).

### Metabolite profiling and quantification of plant bioactive compounds using UHPLC-MS/MS

**Figure 5:**
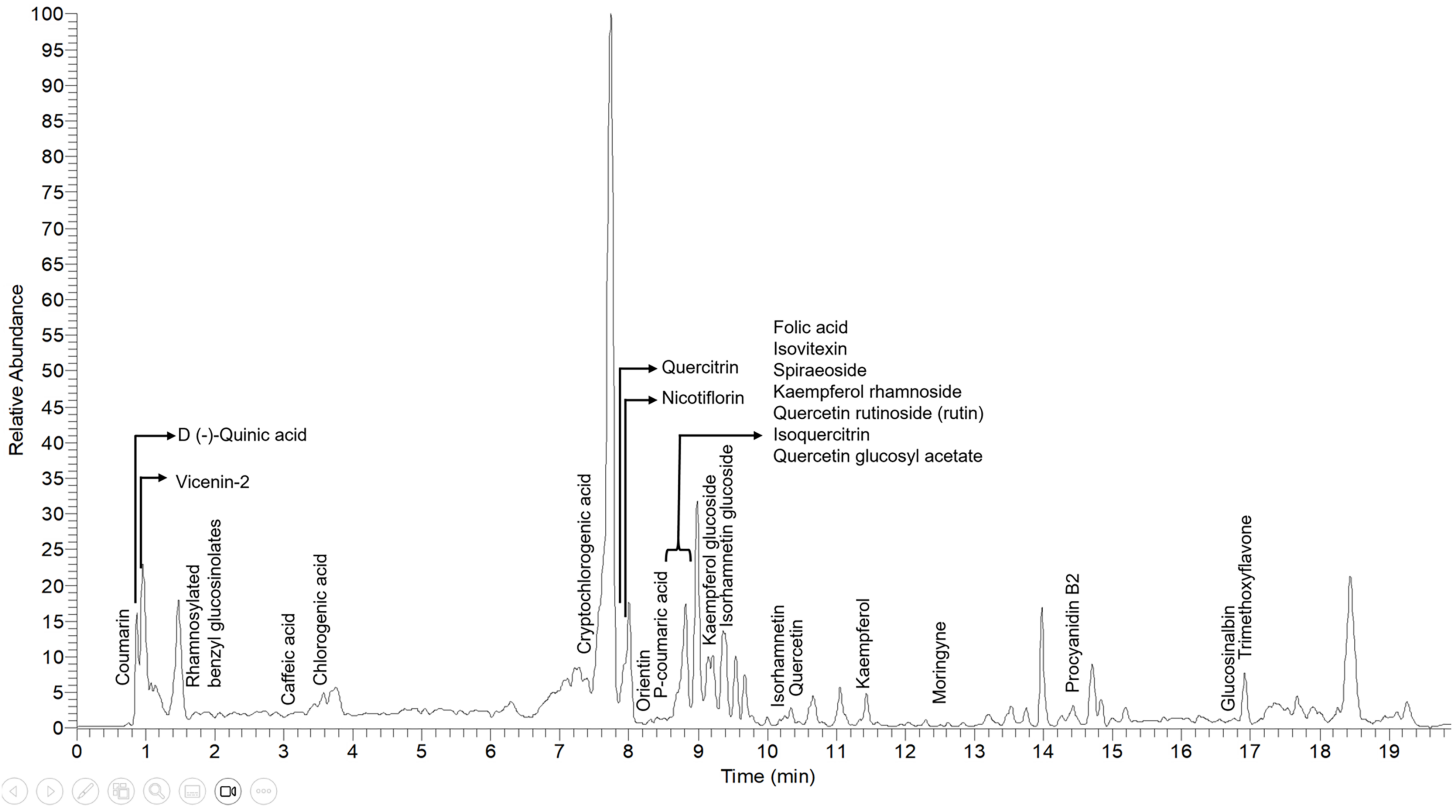
Total ion chromatogram (TIC) of *Moringa oleifera* leaves extract (pooled sample) in negative (−) ionization mode.

From UHPLC-MS/MS analysis, the resulting Total Ion Chromatogram (TIC) is shown in Fig. 5. The TIC reveals broad chemical detection between 0.1 and 20.0 min, with most high-intensity peaks appearing within the 3–15-min range. A total of 27 metabolites were identified from *M. oleifera* leaf extracts across all maturity groups (Table 3). Six metabolites which are kaempferol, quercetin, kaempferol glucoside, quercetin rutinoside, p-coumaric acid, and caffeic acid were confirmed and absolutely quantified using authentic standards (MSI-1), while seven metabolites achieved confident library spectral matches (MSI-2a, match score ≥ 70%) and two of it were supported by the fragment matches from spectral libraries and adduct/RT reasoning (MSI-2b). The remaining entries were classified as MSI-3 (putative class level), where isomeric resolution could not be confirmed but annotated through fragmentation patterns. Overall, from the 27 metabolites, six were absolutely quantified, and 21 metabolites were putatively identified with different MSI classifications.

**Table 3:** Mass spectral characteristics and tentative identification of compounds present 80% ethanolic leaf extract of *M. oleifera*.

No	Retention time, min	Exact mass	Deprotonated molecule, [M-H]-(m/z)	Mass error	MS/MS fragment ions	Metabolites	Molecular formula	Class compound	Identification	MSI levels, Library scoring
1	0.838	146.0368	145.0753	0.96	83.0192, 101.0325, 127.0479	Coumarin	C_9_H_6_O_2_	Phenolics	Putative	*MSI-3*, library matching
2	0.919	192.0634	191.1039	0.96	58.100, 67.400, 70.300, 80.900	D (-)-Quinic acid	C_7_H_12_O_6_	Phenolic acids	Putative	*MSI-2a*, 96.3%
3	0.995	594.1585	593.2928	0.87	297.0711, 325.0720, 335.0633, 353.0717, 383.0779, 413.1167, 473.1163, 485.1098, 503.1221, 547.2747, 557.1413, 575.3552	Vicenin-2	C_27_H_30_O_15_	Flavonoids	Putative	*MSI-3*, library matching
4	1.452	571.1029	570.1833	0.92	225.9480, 259.9687, 331.9899, 374.0546, 390.0317, 406.0266, 408.0423, 422.0579, 424.2003, 450.0529	Rhamnosylated benzyl glucosinolates	C_20_H_29_NO_14_S_2_	Glucosinolates derivatives	Putative	*MSI-2a*, 77.2%
5	3.089	180.04226	179.2911	0.75	132.9386, 136.9010, 150.1936	Caffeic acid*	C_9_H_8_O_4_	Phenolic acids	Quantified	*MSI-1*, library matching
6	3.527	354.0951	353.1282	0.97	173.300, 179.100, 190.500	Chlorogenic acid	C_16_H_18_O_9_	Phenolic acids	Putative	*MSI-3*, library matching
7	7.454	354.0951	353.2495	0.85	109.0304, 134.1229, 135.0459, 135.8258, 137.0221, 155.0359, 155.8535, 161.0225, 173.0453, 179.0350, 191.0556, 238.8018, 284.8653	Cryptochlorogenic acid	C_16_H_18_O_9_	Phenolic acids	Putative	*MSI-2a*, 95.3%
8	7.885	448.1006	447.1469	0.95	161.01489, 247.05663, 269.01028, 357.55563	Quercitrin	C_21_H_20_O_11_	Flavonoids	Putative	*MSI-3*, library matching
9	7.992	594.1585	593.2932	0.87	300.0236, 335.0413, 573.0691	Nicotiflorin	C_27_H_30_O_15_	Flavonoids	Putative	*MSI-3*, library matching
10	8.304	448.1006	447.1996	0.90	285.0488, 285.9653, 297.0556, 299.0421, 299.9790, 309.0391, 325.0762, 327.0629, 328.0126, 328.9071, 337.0564, 339.0616, 339.9998, 341.0139, 357.0717, 358.0159, 358.9066, 369.0756	Orientin	C_21_H_20_O_11_	Flavonoids	Putative	*MSI-2a*, 99.3%
11	8.434	164.16	163.8898	0.27	117.1288, 158.8401, 129.0398	P-coumaric acid*	C_9_H_8_O_3_	Phenolic acids	Quantified	*MSI-1*, library matching
12	8.683	441.1397	440.1633	0.98	352.2885, 394.5964, 397.5489	Folic acid	C_19_H_19_N_7_O_6_	Vitamins/Cofactors	Putative	*MSI-2a*, 79.0%
13	8.693	432.1056	431.1977	0.91	283.058, 293.041, 311.050, 312.054, 323.052, 341.061, 342.062, 353.057, 365.058, 383.072, 413.089	Isovitexin	C_21_H_20_O_10_	Flavonoids	Putative	*MSI-2a*, 93.9%
14	8.759	464.0955	463.1633	0.93	151.0, 300.0, 301.0, 302.0	Spiraeoside	C_21_H_20_O_12_	Flavonoids	Putative	*MSI-3*, library matching
15	8.759	432.10565	431.31857	0.79	255.0982, 256.3233, 431.1810, 432.3161	Kaempferol rhamnoside	C_21_H2_0_O_10_	Flavonoids	Putative	*MSI-3*, library matching
16	8.787	610.521	609.8674	0.65	301.3146, 344.2962, 607.3510, 609.1031	Quercetin rutinoside (rutin)*	C_27_H_30_O_16_	Flavonoids	Quantified	*MSI-1*, library matching
17	8.795	464.0955	463.2187	0.88	151.0494, 151.9679, 179.0329, 255.0425, 255.9785, 272.0024, 272.9251, 300.0397, 301.0162, 301.0424, 302.8185	Isoquercitrin	C_21_H_20_O_12_	Flavonoids	Putative	*MSI-3*, library matching
18	8.977	506.4159	505.2906	1.13	151.00177, 179.99963, 211.0361, 226.02142, 226.03078, 227.03583, 243.02925, 254.02321, 255.02765, 257.03677, 271.02356, 272.00906, 272.02911, 273.03284, 283.02258, 300.02548, 301.03201, 302.03217, 353.09036, 463.08887	Quercetin glucosyl acetate	C_23_H_22_O_13_	Flavonoids	Putative	*MSI-3*, library matching
19	9.131	448.23312	447.2363	1.00	227.0, 255.0, 282.0, 284.0, 285.0	Kaempferol glucoside*	C_21_H_20_O_11_	Flavonoids	Quantified	*MSI-1*, library matching
20	9.216	478.1111	477.2144	0.90	242.2429, 243.0302, 257.0467, 271.0255, 285.0401, 286.0503, 299.0214, 300.0293, 314.044, 315.0536	Isorhamnetin glucoside	C_22_H_22_O_12_	Flavonoids	Putative	*MSI-3*, library matching
21	10.213	316.0583	315.2551	0.80	160.0139, 179.00009, 241.01582	Isorhamnetin	C_16_H_12_O_7_	Flavonoids	Putative	*MSI-2b*, library matching
22	10.464	302.238	301.3203	0.92	120.9556, 180.8438, 187.1749, 190.9658, 255.1022, 300.3669, 301.2993	Quercetin*	C_15_H_10_O_7_	Flavonoids	Quantified	*MSI-1*, library matching
23	11.432	286.239	285.3855	0.85	136.7769, 162.8681, 187.0459, 227.1470, 228.1973, 250.0777, 267.2158	Kaempferol*	C_15_H_10_O_6_	Flavonoids	Quantified	*MSI-1*, library matching
24	12.504	312.1209	311.2965	0.82	191.0708, 223.0970, 293.1025	Moringyne	C_15_H2_0_O_7_	Alkaloids	Putative	*MSI-3*, library matching
25	14.476	578.1424	577.4157	0.73	165.02245, 188.04057, 206.0141, 206.04076, 243.06335, 281.03806, 299.00351, 299.05054, 300.06104, 533.14709	Procyanidin B2	C_30_H_26_O_12_	Flavonoids	Putative	*MSI-2b*, library matching
26	16.835	425.0450	424.4455	0.60	164.0176, 276.0006, 406.0272	Glucosinalbin	C_14_H_19_NO1_0_S_2_	Glucosinolates	Putative	*MSI-3*, library matching
27	16.907	312.0998	311.3481	0.75	184.0244, 185.0263, 211.0297, 247.2149, 311.1969	Trimethoxyflavone	C_18_H_16_O_5_	Flavonoids	Putative	*MSI-3*, library matching

**Note:**

Compound identification confidence was assigned according to the Metabolomics Standard Initiative (MSI). MSI-1: *metabolites quantified; confirmed with internal standard (same-method RT ±0.2 min + MS/MS fragment match); library scores not applicable. *MSI-2a:* putative metabolites *via* library MS/MS fragment match (report match score when available; cutoff ≥70%). *MSI-2b:* putative metabolites through MS/MS fragment match and adduct/RT reasoning without numeric score (list ≥2 fragment match). *MSI-3:* putative metabolites, isomers not uniquely resolved. Library matching indicates tentative match with characteristics MS/MS fragments ions from mass spectral databases.

Flavonoids represented the dominant chemical class, accounting for approximately 59% of all identified metabolites. This group included flavonol aglycones (*e.g*., quercetin, kaempferol, isorhamnetin), glycosylated flavonols (*e.g*., rutin, isoquercitrin, spiraeoside), C-glycosyl flavones (*e.g*., orientin, isovitexin, vicenin-2), and procyanidin B2, a dimeric flavanol. Phenolic compounds, including caffeic acid, chlorogenic acid, p-coumaric acid, and coumarin (a non-flavonoid phenolic), contributed approximately 19%, followed by glucosinolates and their derivatives (*e.g*., rhamnosylated benzyl glucosinolates, glucosinalbin) at 7%. Minor constituents like folic acid and moringyne, each constituting roughly 4%.

The distribution of these metabolites across different leaf age groups exhibited a non-linear, erratic pattern, suggesting that metabolite accumulation is determined by age-dependent and metabolically dynamic processes. As presented in Table 4, the quantified metabolites showed that some reached peak levels during intermediate maturity stages (day-45), while others predominated in either younger (day-30) or older (day-60) leaf extracts. For example, kaempferol glycoside, the highest quantified metabolite, was most abundant in day-45 extracts, followed by day-60 and day-30 (day-45 > day-60 > day-30), indicating enhanced flavonoid biosynthesis during mid-leaf maturation. Conversely, caffeic acid, the least abundant quantified compound, showed its highest levels in day-60 leaves, followed by day-45 and day-30 (day-60 > day-45 > day-30).

**Table 4:** Distribution of quantified metabolites in *M. oleifera* leaf extract for day-30, day-45 and day-60 with limit of detection (LOD) and limit of quantification (LOQ) values.

Quantified metabolite (mg/g)	Day-30	Day-45	Day-60	LOD (mg/mL)	LOQ (mg/mL)
Kaempferol glycoside	25.1985	42.4390	29.4562	0.0045	0.0150
Quercetin rutinoside	0.8429	0.5521	0.8307	0.0020	0.0070
Quercetin	0.4138	0.3758	0.3755	0.0020	0.0060
P coumaric acid	0.0330	0.0227	0.0451	0.0015	0.0050
Kaempferol	0.0198	0.0229	0.0241	0.0030	0.0100
Caffeic acid	0.0109	0.0131	0.0164	0.0025	0.0085

### Multivariate data analysis using SIMCA

**Figure 6:**
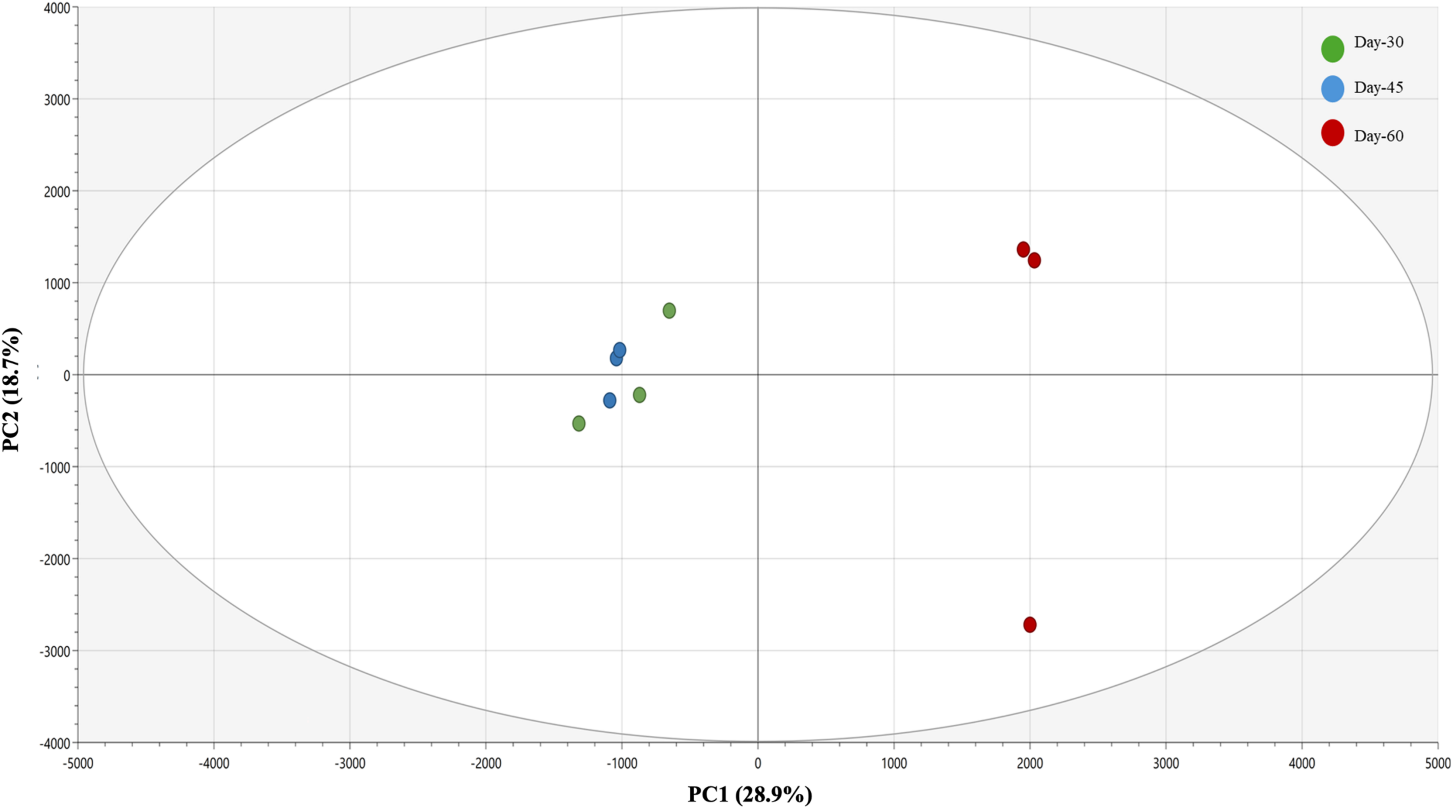
PCA scores plot of UHPLC-MS/MS metabolite data. Samples from day-30 (green), day-45 (blue), and day-60 (red) *M. oleifera* leaves extracts cluster distinctly, with PC1 and PC2 explaining 28.9% and 18.7% of the total variance, respectively.

Unsupervised PCA was conducted to examine the intrinsic variance in metabolite profiles of *M. oleifera* leaf extracts across all stage groups. The PCA scores plot (Fig. 6) showed a clear clustering of extracts following stage groups. PC1 accounted for 28.9% of the variance, while PC2 explained 18.7%, capturing a cumulative 47.6% of the total variance in the dataset. Notably, day-60 samples formed a distinct cluster on the right side of the PCA plot, whereas day-30 and day-45 clustered closer together on the left, indicating that metabolic differentiation becomes more pronounced at later stages of leaf development.

**Figure 7:**
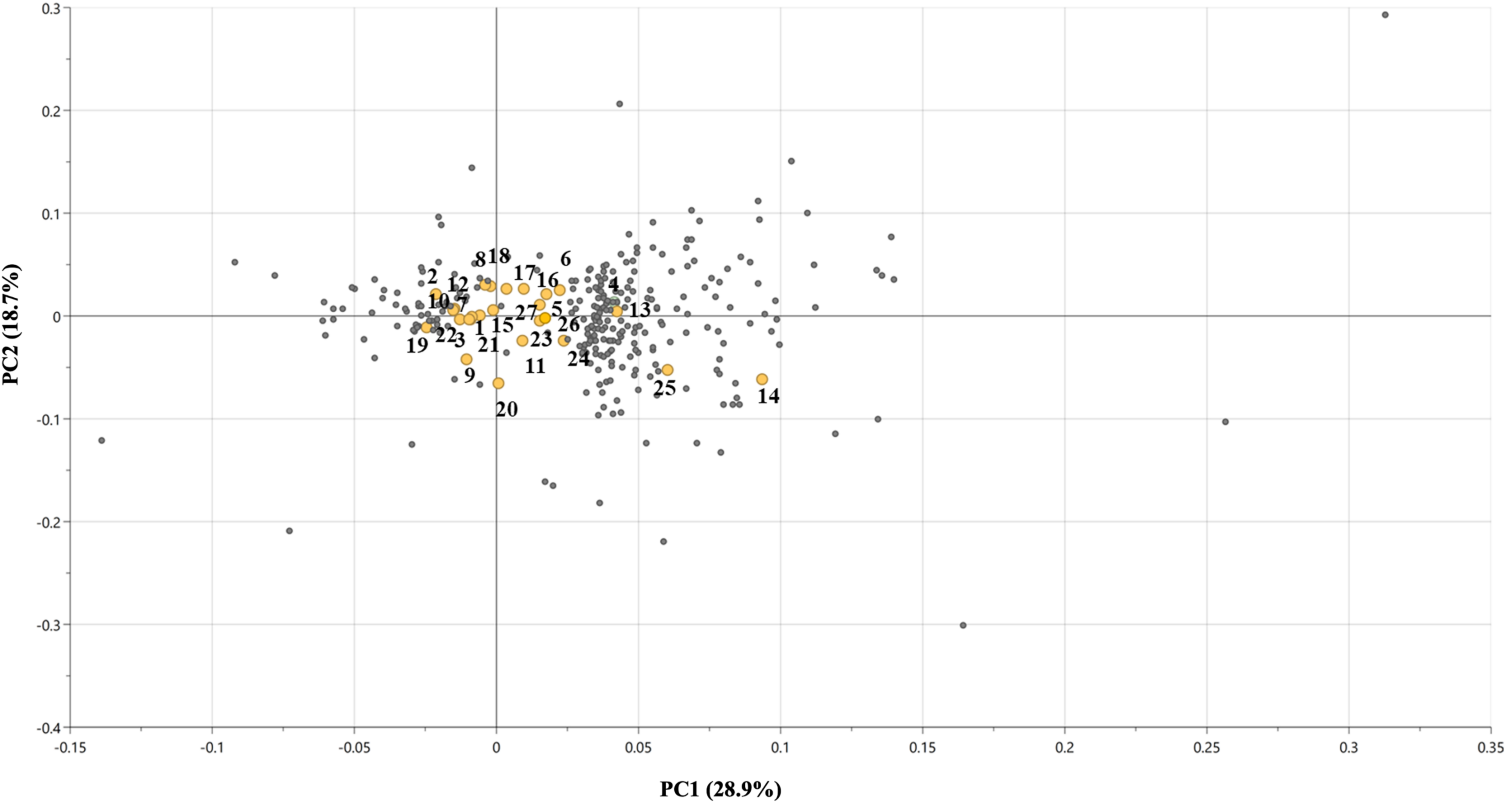
PCA loadings Plot of UHPLC-MS/MS metabolite data. Colored dots represent annotated metabolites contributing to separation along PC1 and PC2 while smaller grey dots represent unknown and unannotated metabolites.

The corresponding PCA loadings plot (Fig. 7) identified key metabolites contributing to the observed separation. Metabolites such as **(16)** quercetin rutinoside, **(17)** quercetin glucoside, and **(25)** procyanidin B2 exhibited high loadings along PC1, implying that these flavonoid derivatives are important distinguishing metabolites of day-60 group. In contrast, metabolites like **(5)** caffeic acid, **(1)** coumarin, and **(2)** quinic acid were more centrally located, suggesting consistent expression across all age groups and minimal contribution to group-level variance.

**Figure 8:**
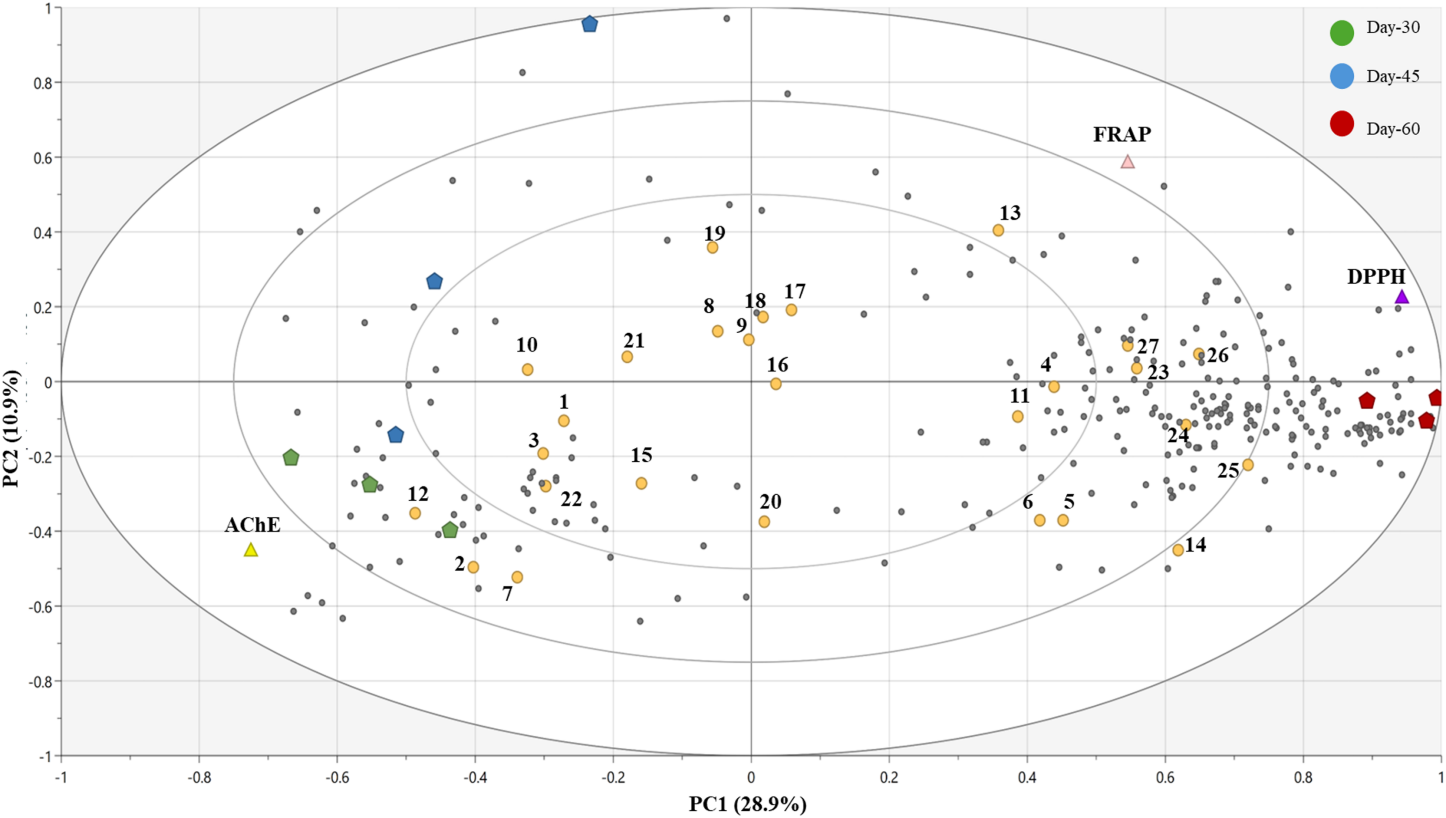
PLS biplot UHPLC-MS/MS metabolite data. Annotated metabolites contributing to group separation and related bioactivities, particularly in day-60 samples. Unknown metabolites shown as grey dots.

Following the PCA results, a PLS biplot model was generated linking the metabolite features to bioactivity readouts (DPPH, FRAP, AChE), emphasizing day-30 and day-60 stages, where the activity contrasts were the largest. PLS-biplot (Fig. 8) showed the distribution of identified and unannotated metabolites with VIP scores >1.0 based on their contribution to the sample separation and influence on model classification. Prominent metabolites included (**14**) spiraeoside, (**17**) quercetin glucoside, (**16**) quercetin rutinoside, (**25**) procyanidin B2, (**6**) chlorogenic acid, (**15**) kaempferol rhamnoside, and (**13**) isovitexin. These metabolites were predominantly enriched in the day-60 group, as demonstrated by their localisation to the day-60 cluster in the biplot, suggesting late-stage accumulation of flavonoid derivatives.

Correlation with bioactivity indices, as illustrated in the PLS biplot, revealed that several metabolites were spatially aligned with functional outcomes. Metabolites such as (**16**) quercetin rutinoside, (**17**) isoquercitrin, (**18**) quercetin glucosyl acetate, (**19**) kaempferol glucoside, and (**23**) kaempferol clustered in the same direction as the DPPH and FRAP vectors, indicating a strong positive association with antioxidant capacity. Likewise, (**25**) procyanidin B2 and (**26**) glucosinalbin also aligned positively with FRAP and DPPH, suggesting their functional role in elucidating antioxidant responses. In contrast, (**1**) coumarin and (**12**) folic acid was directionally aligned with the AChE inhibition vector, implying a potential neuromodulatory or enzyme inhibitory role. Interestingly, some polyphenols such as (**5**) caffeic acid and (**6**) chlorogenic acid were not tightly clustered near any single bioactivity vector but remained within the reflecting region of FRAP and DPPH, which may indicate moderate antioxidant relevance.

For MVDA model validation, unsupervised PCA resolved clear group separation (Fig. 7) across maturity stages, indicating that the major variance is stage-related without supervision. The two-component PCA model explained 47.6% of the total X-variance (R^2^X [1] = 0.289; R^2^X [2] = 0.187; R^2^X(cum) = 0.476), indicating that nearly half of the metabolomics variation was captured by these principal components. The Q^2^ (cum) was slightly negative (−0.124), suggesting limited predictive ability, likely due to the small sample size, with high variable features. However, as this is for unsupervised determination targeted for pattern recognition rather than prediction, the low value is acceptable (Debik et al., 2022). For the PLS biplot model that linked metabolite features to bioactivity data, it achieved a strong fit (R^2^Y(cum) ≈ 0.91) but low predictive ability (Q^2^ cum = 0.0599, 7-fold CV). In addition, permutation testing (*n* = 200) yielded near-zero R^2^/Q^2^ intercepts, suggesting that the observed fit is not due to chance. However, the modest balance accuracy and cross-validated confusion matrix were consistent with the low Q^2^. Thus, the PLS-DA model was interpreted as exploratory, using only VIP > 1 to nominate candidates. Consequently, the biological conclusions were grounded on quantified/standard-confirmed metabolites and consistent bioactivity assay trends.

## Discussion

The findings in this study are consistent with those reported by Nobossé, Fombang & Mbofung (2018), who observed significantly higher total flavonoid content (*p* < 0.05) in day-60 *M. oleifera* leaves (1.82 ± 0.07 g CE/100 g DM) compared to day-30 leaves (1.40 ± 0.04 g CE/100 g DM). Similarly, the total phenolic content was lowest in day-30 leaves (3.32 ± 0.37 g CE/100 g DM) and increased significantly by day-60 (3.97 g CE/100 g DM). The variation in results between their study and our study may be attributed to differences in extraction methods in which they have used aqueous, ethanol, and methanol extractions, while this study used only ethanol. In addition, the study by Sreelatha & Padma (2009) supports these observations, showing an increase in both total flavonoid and phenolic contents with increasing maturity stages of *M. oleifera* leaves.

The IC_50_ results from the DPPH assay in this study are comparable to findings by Nobossé, Fombang & Mbofung (2018), who studied *M. oleifera* leaves from Cameroon using the same ethanol extraction method. They reported that day-60 leaves had antioxidant activity close to that of ascorbic acid (0.1 mg/mL). Sreelatha & Padma (2009) also found that mature *M. oleifera* leaves had higher antioxidant activity than young leaves (IC_50_: 18.15 ± 0.92 *vs* 19.12 ± 0.75), attributing this to increased phenolic and flavonoid content at later growth stages (Sreelatha & Padma, 2009). The rise in antioxidant potential with increasing leaf maturity in both studies may be due to the accumulation of bioactive compounds such as flavonoids and phenols, which are synthesized progressively during leaf development (Kong et al., 2017).

For ferric reducing power, we found no significant differences (*p* > 0.05) between the three maturity levels of *M. oleifera* leaf extract and all values were lower than that of the ascorbic acid standard. This contrasts with Pakade, Cukrowska & Chimuka (2013), who found *M. oleifera* leaves from South Africa exhibited stronger ferric reducing power than ascorbic acid when using aqueous extraction. The difference could stem from geographical variation in phytochemical content or the use of ethanol extraction in the present study *vs* aqueous in theirs. Nobossé, Fombang & Mbofung (2018) reported that FRAP activity was highest in day-45 leaves and lowest in day-60 leaves for all extraction methods (methanol, ethanol, and aqueous). Their results differ from the current study, where the lowest FRAP activity was seen on day-30. These inconsistencies may be attributed to regional differences in phytochemical profiles. They also observed a moderate correlation (r = 0.59) between chlorophyll content and FRAP activity. Hence, Malaysian *M. oleifera* leaves, particularly on day-30, may contain lower levels of chlorophyll and related phytochemicals, resulting in weaker ferric reducing power.

The finding in our anticholinesterase inhibition activity aligns with a previous study that assessed AChE inhibitory activity using various parts of the *M. oleifera* plant (seeds, leaves, roots, flowers, stems) extracted with methanol, ethanol, and aqueous solvents (Nwidu et al., 2018). In that study, the ethanol extract of the leaves exhibited 50% AChE inhibition (IC50: 0.2105), which is approximately 14% higher than the inhibition observed in this study. However, the earlier study did not control for specific leaf maturity stages and used a test concentration of 200 µg/mL for the highest inhibition. Anticholinesterase activity is commonly associated with bioactive compounds such as alkaloids and terpenoids, which are abundant in traditional medicinal plants including *M. oleifera* (Mukherjee et al., 2007). In addition, other non-alkaloid metabolites such as phenolics, flavonoids and coumarin-type compounds are also known as the reversible AChE inhibitors (Abu-Aisheh et al., 2019; Khan et al., 2019; Szwajgier, 2015).

Previous research indicates that alkaloids are more efficiently extracted with ethanol, whereas terpenoids are better extracted with aqueous solvents (Adekanmi, Adekanmi & Adekanmi, 2020). However, in the present work, the untargeted UHPLC-MS/MS performed in negative ESI mode is less sensitive to alkaloids, making it difficult to confirm their presence in the leaf extracts. Hence, it is more plausible that the observed AChE inhibition activities are attributed to the non-alkaloid metabolites. Indeed, the PLS biplot model presented alignment of coumarin and folic acid with the AChE vector, thus supporting the likelihood of their contribution to enzyme inhibition in day-30 extracts. Nonetheless, the confounding basis remains exploratory, and future studies are necessary to confirm the neuroprotective potential of young *M. oleifera* leaf (day-30).

The chemical diversity of *M. oleifera* leaves highlights their potential contribution to antioxidant and neuroprotective bioactivities (Leone et al., 2016; Mbikay, 2012; Vergara-Jimenez, Almatrafi & Fernandez, 2017). Besides chemical classification, some metabolites were known to have a scientifically proven or predicted biological relevance. From the flavonoids class, kaempferol, quercetin, and their glycosides such as kaempferol glucoside, quercetin rutinoside (rutin), spiraeoside, and isoquercitrin are well-documented for their strong antioxidant, anti-inflammatory, and neuroprotective properties (Choy et al., 2019; Manach et al., 2004). C-glycosyl flavones such as orientin, isovitexin, and vicenin-2, which has been detected consistently in all stage groups, are well known for their chemical stability, conferred by the C–C glycosidic bond, and UV-protective properties. Vicenin-2, has been shown to accumulate in upper leaf tissues, acting as a natural UV filter (Silva et al., 2014). Additionally, orientin and vicenin-2 have demonstrated radioprotective and antioxidant effects by modulating oxidative stress pathways (Uma Devi et al., 2000), while their glycosylated structures ensure high metabolic stability and bioactivity in plants (Rauter, Lopes & Martins, 2007). The detection of caffeic acid, chlorogenic acid, and quinic acid supports active flux through the phenylpropanoid and shikimate pathways (Dixon & Paiva, 1995). While coumarin appeared in lower abundance, its hepatoprotective, anticoagulant, and antimicrobial activities are well established (Rauter, Lopes & Martins, 2007). The identification of glucosinalbin and rhamnosylated benzyl glucosinolates reinforces the functional sulfur-rich profile of *M. oleifera*, contributing to detoxification and pathogen defense (Fahey, 2005). Moreover, procyanidin B2, a proanthocyanidin flavanol, is recognized for its free radical scavenging and cardiovascular benefits (Santos-Buelga & Scalbert, 2000). Compounds such as moringyne and trimethoxyflavone, although less studied, may represent novel bioactive compound unique to *M. oleifera*, prompting for future investigation.

The distribution of these metabolites across different leaf age groups exhibited a non-linear, erratic pattern, suggesting that metabolite accumulation is determined by age-dependent and metabolically dynamic processes. These variable distribution trends likely reflect the complex and multifactorial regulation of secondary metabolism throughout plant development, which may not follow a linear or monotonic progression. The biosynthesis of phenolic compounds, including flavonoids and hydroxycinnamic acids, is often regulated by internal developmental signals and external environmental stimuli (Dixon & Paiva, 1995). Such compounds function in stress responses, particularly against oxidative stress and UV damage, and their accumulation may vary with the physiological demands at different growth stages (Manach et al., 2004; Panche, Diwan & Chandra, 2016). In addition, Managa, du Toit & Prinsloo (2021) reported that moderate harvesting frequencies increased levels of chlorogenic acid and flavonoid glycosides, suggesting that optimal biosynthesis occurs within a specific physiological condition. Similarly, environmental parameters such as altitude and seasonal variation, as well as processing and storage, have been shown to significantly affect the accumulation of key metabolites in *M. oleifera* (Ralepele et al., 2021).

Interestingly, this non-linear metabolite pattern contrasts with the more linear trend observed in the bioactivity assays across the different stage groups. This discrepancy may be resulted from the limited subset of metabolites that were confidently identified and quantified, which likely does not encompass all bioactive constituents contributing to the bioactivities. Hence, untargeted metabolite peak areas extracted through MS-DIAL chromatogram processing were incorporated into downstream MVDA by using SIMCA software. PLS biplot, a regression model was applied to correlate non-targeted metabolite peak area with the respective bioactivities such as DPPH, FRAP, AChE inhibition obtained priorly. This integrative metabolomics approach enables identification of additional, potentially unannotated compounds that contribute to the observed biological effects and may serve as predictive biomarkers for functional efficacy.

The clustering of structurally related compounds such as quercetin glycosides, kaempferol derivatives, and chlorogenic acids reflects coordinated biosynthetic regulation, consistent with known age-dependent metabolic association in flavonoid and phenolic pathways (Managa, du Toit & Prinsloo, 2021; Panche, Diwan & Chandra, 2016). Notably, the loadings plot also included small grey dots, representing unannotated or unknown metabolite features detected in the untargeted profiling. These features were retained during MS-DIAL preprocessing based on reproducibility and peak area, despite lacking high-confidence library matches. While these unknown compounds were unidentified, their distribution in PCA especially those located at the periphery of the loading plot suggests that some may contribute significantly to the variance observed across maturity stages. Such unknowns are a common occurrence in metabolomics studies and represent potential novel biomarkers or uncharacterized phytochemicals (Scalbert et al., 2014).

Unsupervised PCA reflected a genuine age-related distribution, however, although PLS model fit was good with high R^2^Y, the low Q^2^ denotes a limited predictive power and likely a biological overlap between adjacent maturity stages. Thus, it is used exploratorily for feature nomination rather than hard classification. Permutation testing supported that the observed structure is not random, but mechanistic inferences are associated to verified metabolites, and PLS biplot is regression model that linked the metabolites signals to bioactivities. Within this framework, the distribution and clustering of spiraeoside, quercetin glucoside, quercetin rutinoside, procyanidin B2, chlorogenic acid, kaempferol rhamnoside, and isovitexin reflect their enrichment in mature (day-60) *M. oleifera* leaves, and these compounds have been previously associated with antioxidant and stress-response activities in plants (Manach et al., 2004; Managa, du Toit & Prinsloo, 2021). This suggests that the late-stage accumulation of flavonoid derivatives may enhance the antioxidant profile of mature leaves.

The spatial alignment of metabolites such as quercetin rutinoside, isoquercitrin, quercetin glucosyl acetate, kaempferol glucoside, and kaempferol with DPPH and FRAP vectors underscores their strong positive association with antioxidant activity. These flavonoid derivatives are well known for their radical scavenging properties, further supporting their contribution to the antioxidant potential observed in day-60 extracts (Choy et al., 2019; Manach et al., 2004). Similarly, procyanidin B2 and glucosinalbin also also aligned positively with FRAP and DPPH, suggesting their functional role in elucidating antioxidant responses. Coumarin and folic acid, which aligned with the AChE inhibition vector, suggest possible roles in neuromodulation or enzyme inhibition. Coumarin-derived compounds have demonstrated acetylcholinesterase inhibitory activity and central nervous system-modulating effects (Venugopala, Rashmi & Odhav, 2013), while folic acid has been shown to reduce AChE activity in the brains of hypothyroid rats, improving their performance in memory tasks through Morris water maze and passive avoidance test evaluation (Amirahmadi et al., 2021). The moderate positioning of caffeic acid and chlorogenic acid near antioxidant vectors suggests a potential, though not dominant, role in antioxidant mechanisms.

These findings support that stage-related metabolic differentiation in *M. oleifera* leaves is largely driven by the accumulation of phenolic acids, flavonoid glycosides, and sulfur-containing secondary metabolites, with mid- to late maturity stages (day-45 to day-60) showing greater bioactivity potential. However, this study acknowledged the limitations of adjacent class overlap and limited sample size that likely suppress the classification of Q^2^, avoiding over-interpretation of PLS class accuracy. Future work should improve predictability through larger cohorts, external test sets, or repeated double cross-validation, variable reduction to confirmed metabolites, and class-balanced designs.

## Conclusions

This study has profiled the metabolome of *M. oleifera* leaves by integrating bioassays with untargeted UHPLC-MS/MS and chemometrics analysis across leaf maturity (day-30, day-45, and day-60). Day-60 extracts showed the highest TPC and TFC values, with the strongest antioxidant capacity *via* DPPH and FRAP assays, whereas AChE inhibition peaked at day-30 extracts. Metabolomics annotated a total of 27 metabolites, which predominantly are flavonoid class, while PCA analysis resolved the maturity-linked metabolite clustering and highlighted the discriminants alongside the high-VIP unknowns. PLS biplot analysis has demonstrated contributions of quercetin, chlorogenic acid, and procyanidin B2 to antioxidant capacity, and has suggested non-flavonoid metabolites as contributors to AChE activities. The results have thus indicated day-60 extracts as the optimal stage for antioxidant-related applications, and day-30 extracts for neuroprotective potential. However, this study also display limitations of using absolute Da tolerances in MS-DIAL parameters while processing ion-trap data, which may influence low-abundance feature calls, and a limited sample size that likely suppresses the classification of Q^2^ for the PLS model. Hence, future studies with larger cohorts, external test sets, and refined processing are suggested to improve predictability and annotation confidence. Collectively, the integration of metabolomics, bioassays, and chemometrics analysis has provided a critical framework for data-driven harvest timing, quality assessment, functional ingredient discovery, and rational development of *M. oleifera* nutraceuticals.

## Supplemental Information

10.7717/peerj.20938/supp-1 Supplemental Information 1 Feature data table for peak area data with filtered VIP (>1), and bioactivities data for multivariate data analysis (MVDA) using SIMCA.

## Additional Information and Declarations

### Data Availability

The following information was supplied regarding data availability:

The raw data is available in the Supplemental File.
